# A Novel Gravity Compensation Method for High Precision Free-INS Based on “Extreme Learning Machine”

**DOI:** 10.3390/s16122019

**Published:** 2016-11-29

**Authors:** Xiao Zhou, Gongliu Yang, Qingzhong Cai, Jing Wang

**Affiliations:** 1 School of Instrument Science and Opto-Electronics Engineering, Beihang University, Beijing 100191, China; yanggongliu@buaa.edu.cn (G.Y.); qingzhong_cai@buaa.edu.cn (Q.C.); by1217133@buaa.edu.cn (J.W.); 2 Science and Technology on Inertial Laboratory, Beihang University, Beijing 100191, China

**Keywords:** gravity compensation, error modelling, extreme learning machine (ELM), high precision free-INS

## Abstract

In recent years, with the emergency of high precision inertial sensors (accelerometers and gyros), gravity compensation has become a major source influencing the navigation accuracy in inertial navigation systems (INS), especially for high-precision INS. This paper presents preliminary results concerning the effect of gravity disturbance on INS. Meanwhile, this paper proposes a novel gravity compensation method for high-precision INS, which estimates the gravity disturbance on the track using the extreme learning machine (ELM) method based on measured gravity data on the geoid and processes the gravity disturbance to the height where INS has an upward continuation, then compensates the obtained gravity disturbance into the error equations of INS to restrain the INS error propagation. The estimation accuracy of the gravity disturbance data is verified by numerical tests. The root mean square error (RMSE) of the ELM estimation method can be improved by 23% and 44% compared with the bilinear interpolation method in plain and mountain areas, respectively. To further validate the proposed gravity compensation method, field experiments with an experimental vehicle were carried out in two regions. Test 1 was carried out in a plain area and Test 2 in a mountain area. The field experiment results also prove that the proposed gravity compensation method can significantly improve the positioning accuracy. During the 2-h field experiments, the positioning accuracy can be improved by 13% and 29% respectively, in Tests 1 and 2, when the navigation scheme is compensated by the proposed gravity compensation method.

## 1. Introduction

Precise navigation is an essential factor of modern carriers. Nowadays, most modern vehicles and aircraft depend on the Global Positioning System (GPS) for position update as they navigate. GPS utilizes the signals from navigation satellites to realize high precision locating. However, normally, GPS cannot work when the satellite signals are not available due to physical blockage, such as inside a cave or under water. The inertial navigation system utilizes the laws of Newtonian physics to realize autonomous navigation worldwide in all weather conditions. Due to INS’s inherent character, it can overcome the disadvantages of GPS and widely serve in all branches of military and many civil applications [1,2].

With the rapid improvement of the inertial instruments (accelerometers and gyros), gravity compensation in INS has already become an important way to further improve the navigation accuracy. Estimating the gravity field for navigation with INS is known as gravity compensation. In other words, it uses the position provided by INS to estimate the gravity disturbance vector δg [3].

To achieve high accuracy for INS navigation, there are mainly three feasible ways for gravity compensation [4]. First, the traditional method obtains the gravity disturbance on the trajectory using a gravitational gradiometer that senses the gradient of the potential field [5]. This case depends on augmenting the INS with a sensitive instrument that has a long history of technology development, but very little operational experience. The second method is to use existing gravity field models (like DQM2000, GFZ97, EGM2008, etc.) and perform compensation on the location results directly by calculating the position errors with the gravity disturbance given [6]. However, the accuracy of these models cannot satisfy the requirements of high precision INS, especially in rough topography, such as mountains, plateaus and oceanic trenches [7]. The third way is to obtain the gravity disturbance using the interpolation method based on measured gravity data on the geoid, then to process the gravity disturbance to the height where INS has an upward continuation [4,8]. In recent years, the most popular interpolation methods used in the geodetic and geophysical communities have been the inverse distance weighted (IDW) interpolation method and the bilinear interpolation method [9,10]. A relationship exists between the position point, and the value of gravity data is different in different areas: linearity in a plain area; nonlinearity in a rough topological area. The above two interpolation methods can achieve a high accuracy when applied in a plain area, yet have bad performance for areas where fierce gravity variation exists, especially in rugged mountain regions. In this paper, a novel gravity disturbance estimation method that uses extreme learning machine (ELM) is proposed to solve this problem. ELM has extensive applications because of its simple structure and mature technology. It provides a new approach for the nonlinear approximation. This motivates this paper to apply ELM to estimate the gravity disturbance on the trajectory [11]. The ELM-based gravity disturbance estimation algorithm is utilized in the training process to establish the prediction model, with the carrier position (longitude and latitude) that the INS provided as input and the gravity disturbance on the geoid as output, then processes the obtained gravity disturbance to the height (provided by the altimeter) where INS has an upward continuation [12,13]. Finally, the estimated gravity disturbance on the trajectory is compensated in the INS error equations incorporated with gravity disturbance to restrain the error propagation in INS. In this paper, numerical tests and field experiments were carried out to verify the accuracy and effectiveness of the proposed gravity compensation method.

The paper is organized as follows: in Section 2, the error analysis of the INS solution incorporated with gravity disturbance is proposed; in Section 3, a brief review of the artificial neural network (ANN) is introduced; in Section 4, the theory and framework of the ELM-based gravity disturbance compensation method are proposed; in Section 5, the numerical tests are designed to prove the accuracy and superiority of ELM-based gravity disturbance estimation method; in Section 6, field experiments in a city area and a mountain area are presented. Finally, Section 7 concludes the paper.

## 2. Error Analysis of INS Solution Considering Gravity Disturbance

### 2.1. Definition of Gravity Disturbance Vector

**Figure 1:**
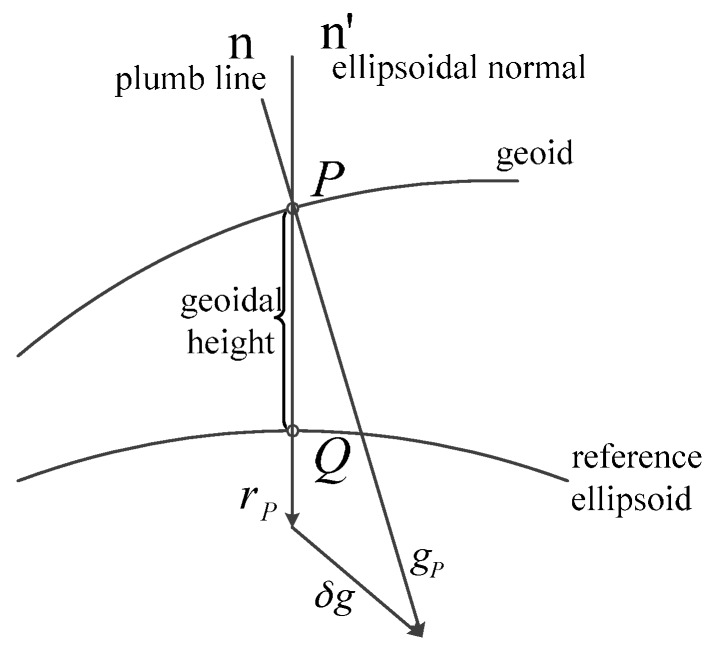
Description of the gravity disturbance vector.

The gravity disturbance vector is the vector difference between the actual gravity and the normal gravity on the same point in space (Figure 1). It is divided into two parts: the tangential component (vertical deflection) and the orthogonal component (gravity anomaly) [14].

In Figure 1, n is the plumb line on the geoid and perpendicular to the geoid, n’ is the ellipsoidal normal on the reference ellipsoid and perpendicular to the reference ellipsoid. Suppose the gravity vector *g_p_* and the normal gravity vector *γ_p_* are at the same point *P*. The gravity disturbance vector *δg* is defined as their difference:

(1) $$\delta g=g_{P}-\gamma_{P}$$

**Figure 2:**
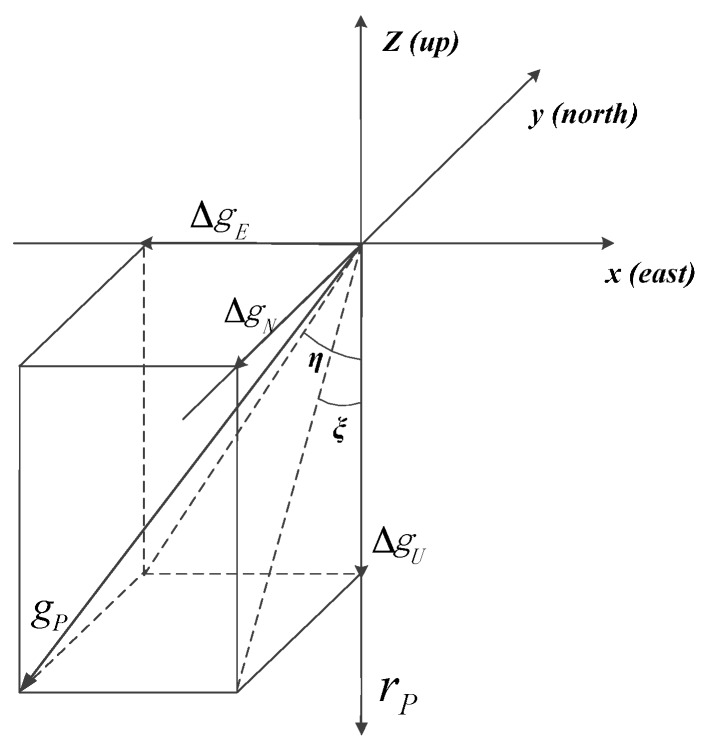
The deflection of the vertical.

$g_{P}$ and *γ_p_* are different in value and direction. The angle between the projection of $g_{P}$ on the north plane and *γ_p_* is defined as ζ, and the angle between the projection of $g_{P}$ on the east plane and *γ_p_* is defined as η. ζ and η together are defined as deflections of the vertical (DOVs), as shown in Figure 2 [14].

(2) $$\delta g=[\Delta g_{E}\Delta g_{N}\Delta g_{U}]$$

The gravity disturbance δg can be decomposed into Cartesian coordinate system: in the east direction, it is $\Delta g_{E}$; in the north direction, it is $\Delta g_{N}$; and in the vertical direction, it is $\Delta g_{U}$. In the gravity database, the gravity anomaly ($\Delta g_{U}$) and DOVs (ζ,η) are given. $\Delta g_{N}$ and $\Delta g_{E}$ are calculated by the following equation:

(3) $$\left\{ \begin{array}{l} \Delta g_{N}=-\gamma_{0}\zeta \\ \Delta g_{E}=-\gamma_{0}\eta \end{array}\right.$$

In Equation (3), $\gamma_{0}$ is the value of normal gravity.

### 2.2. INS Error Equations Considering Gravity Disturbance

The INS error equations when incorporated with gravity disturbance can be defined as follows [15,16]:

(4) $$\begin{array}{l} \delta {\dot{V}}^{n}=-\phi^{n}\times f^{n}+C_{b}^{n}(\delta K_{A}+\delta A)f^{n}+\delta V^{n}\times (2\omega_{ie}^{n}+\omega_{en}^{n})\\ +V^{n}\times (2\delta \omega_{ie}^{n}+\delta \omega_{en}^{n})+\nabla^{n}+\delta g^{n} \end{array}$$

(5) $$\left\{ \begin{array}{l} \delta \dot{L}=\frac{\delta V_{N}}{R_{M}+h}\\ \delta \dot{\lambda }=\frac{\delta V_{E}}{R_{N}+h}\sec\;L+\delta L\frac{V_{E}}{R_{N}+h}\tan\;L\;\sec\;L\\ \delta \dot{h}=\delta V_{U} \end{array}\right.$$

(6) $$\dot{\phi }=\phi \times \omega_{in}^{n}+\delta \omega_{in}^{n}-C_{b}^{n}(\delta K_{G}+\delta G)\omega_{ib}^{b}+\varepsilon^{n}$$

where $V^{n}$ is the velocity in the navigation frame, $\delta V^{n}$ is the velocity error in the navigation frame, $\delta {\dot{V}}^{n}$ is the differential form of $\delta V^{n}$, $\phi^{n}$ is the attitude error, $f^{n}$ is the specific force expressed in the navigation frame, $C_{b}^{n}$ is a direction cosine matrix used to transform the body acceleration vector into the navigation frame, $\delta K_{A}$ and δA are the scale coefficient error and the installation angle error of the accelerometer, respectively, $\nabla^{n}$ is the accelerometer bias expressed in the navigation frame, $\delta g^{n}$ is the gravity disturbance in the navigation frame, L,λ,h are the current latitude, longitude and altitude of the body, δL,δλ,δh denote the latitude error, longitude error and altitude error, respectively, $\delta \dot{L},\delta \dot{\lambda },\delta \dot{h}$ are the differential forms of δL,δλ,δh, $\delta V_{N},\delta V_{E},\delta V_{U}$ are the velocity errors of the north, east and vertical directions, respectively, $R_{M}$ and $R_{N}$ denote the meridian radius and prime vertical radius, respectively, $\delta K_{G}$ and δG are the scale coefficient error and installation angle error of the gyro, respectively, and $\varepsilon^{n}$ is the gyro drift in the navigation frame; $\delta \omega_{in}^{n}$ can be expressed as follows:

(7) $$\delta \omega_{in}^{n}=\delta \omega_{ie}^{n}+\delta \omega_{en}^{n}$$

where $\omega_{ie}^{n}$ and $\omega_{en}^{n}$ are the Earth’s rotation rate and the navigation frame’s rotation with respect to the Earth, respectively; both are expressed in the navigation frame. Their computational formulas are defined as follows:

(8) $$\delta \omega_{en}^{n}=\left[\begin{array}{c} -\frac{\delta V_{N}}{R_{M}+h}\\ \frac{\delta V_{E}}{R_{N}+h}\\ \frac{\delta V_{E}\tan\;L}{R_{N}+h}+\delta L\frac{V_{E}\sec^{2}\;L}{R_{N}+h} \end{array}\right]$$

(9) $$\delta \omega_{ie}^{n}={\left[0\;\;-\delta L\omega_{ie}\sin\;L\;\;\delta L\omega_{ie}\cos\;L\right]}^{T}$$

From Equation (4) to Equation (9), we can see that the accelerometer’s output error $(\delta K_{A}+\delta A)f^{n}+\nabla^{n}$, velocity error $\delta V^{n}$ and gravity disturbance $\delta g^{n}$ are the main error sources causing INS velocity error $\delta V^{n}$. The position errors of INS are mainly caused by velocity error $\delta V^{n}$ and the coupling error between velocity and position.

According to the above analysis, gravity disturbance $\delta g^{n}$ firstly influences INS velocity accuracy through Equation (4) and then affects INS position and attitude accuracy by Equations (5) and (6). With the high-precision inertial sensors being available, errors due to gravity disturbance are equal to or even more than the errors caused by inertial sensors, so the influence of gravity disturbance on INS cannot be ignored and must be compensated to further improve the navigation accuracy of INS.

**Figure 3:**
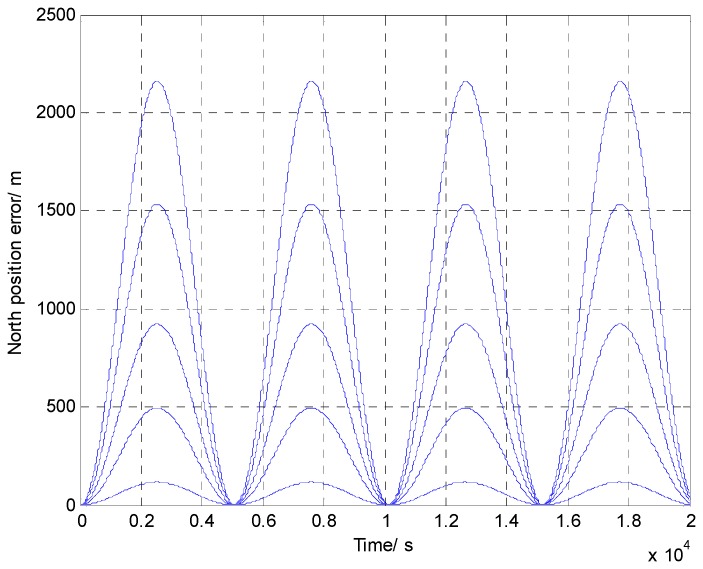
The north position error caused by gravity disturbance.

To better illustrate the effect of gravity disturbance on INS error propagation, we calculated INS north position errors caused by different values of north-south gravity vertical deflection ζ as an example using Equation (5); the results are shown in Table 1 and Figure 3.

From Table 1 and Figure 3, we can draw the conclusion that the position error of the north channel caused by gravity disturbance presents periodic variation on the Schuler cycle (about 84.4 min), and the amplitude is proportional to the value of the horizontal component of gravity disturbance. Therefore, the effect of gravity disturbance for high-precision INS cannot be ignored, and effective measures must be taken to compensate it for achieving a better navigation solution of INS.

## 3. Brief Review of Artificial Neural Networks

**Figure 4:**
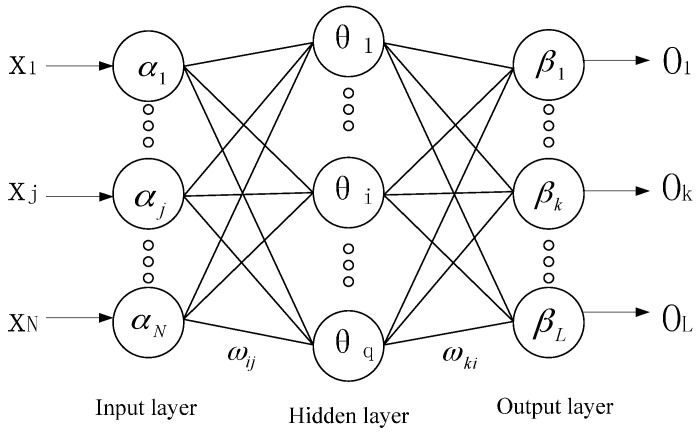
Structure of an artificial neural network.

Nowadays, artificial neural networks (ANNs) have been widely used in most areas. ANNs are computational models that simulate the process of the human brain, and the structure of an artificial neuron is much simpler than a biological neuron. A neural network includes a large number of inter-connected neurons in the input and hidden layers that join to all neurons in the output layer (which is called a full synapse network). Each neuron can do some simple computation, such as summation, subtraction and multiplication. Figure 4 presents the structure of an artificial neural network [17,18,19].

In Figure 4, $X_{1}\cdots X_{N}$ and $O_{1}\cdots O_{L}$ are the input and output of the artificial neural network, respectively; $\alpha_{1}\cdots \alpha_{N}$ are the neuron nodes of the input layer; $\theta_{1}\cdots \theta_{q}$ are the neuron nodes of the hidden layer; $\beta_{1}\cdots \beta_{L}$ are the neuron nodes of the output layer; $\omega_{ij}$ is the connecting weight between the input layer and hidden layer; and $\omega_{ki}$ is the connecting weight between the hidden layer and output layer.

ANN is a nonlinear statistical data modeling or decision-making method, so it can be used to model complex relationships between the input and output or to find patterns in data. ANN has the abilities of being data-driven, having self-learning and self-adaption; meanwhile, it has strong capabilities of anti-jamming. Therefore, the neural network-based gravity disturbance estimation method is proposed in this paper.

## 4. The Theory and Framework of the ELM-Based Gravity Disturbance Compensation Method in INS

In recent years, ELM has become a hot research topic for machine learning and artificial intelligence. Compared with other traditional artificial learning methods, ELM has its own advantages, which are described as follows [11,12]:

(1) No parameters need to be tuned except the predefined network structure;
(2) ELM is capable of faster learning, and most trainings can be completed quickly;
(3) ELM can achieve a high generalization performance;
(4) ELM has a wide selection range of activation functions that are all piecewise continuous functions that can be used as activation functions.

Because of ELM’s many advantages, in this paper, we use ELM to estimate the gravity disturbance on the trajectory based on measured gravity on the geoid and then process the gravity disturbance on the geoid to the height where INS has an upward continuation. Finally, the estimated gravity disturbance on the trajectory is compensated in the INS error equations to restrain the error propagation in INS.

### 4.1. Extreme Learning Machine

The mathematical model of the ELM is described as below [20,21]:

Input and output: Consider that we have a training set ${\left\{\left(x_{i},t_{i}\right)\right\}}_{i=1}^{N}$ with N distinct examples, where $x_{i}={\left[x_{i1},x_{i2},x_{i3},...,x_{in}\right]}^{T}$ has n inputs and $t_{i}={\left[t_{i1},t_{i2},t_{i3},...,t_{im}\right]}^{T}$ has m outputs. Here, we define each input $x_{i}$ to be composed of longitude and latitude, described as $x_{i}=[\lambda_{i}\;L_{i}]$, and the output $t_{i}$ is the gravity disturbance on the geoid, described as $t_{i}=[\Delta g_{Ei}\;\Delta g_{Ni}\;\Delta g_{Ui}]$.

Symbols in the network: Assume that l is the number of hidden neurons, ω is the l × n input weight matrix connecting the i−th hidden neuron and the input neurons where $\omega_{j}={\left[\omega_{j1},\omega_{j2},\omega_{j3},...,\omega_{jn}\right]}^{T}$, $b_{j}$ is the bias for each single hidden neuron, b is the l × 1 biases vector for hidden neurons, β is the l × m output weight matrix connecting the i−th hidden neuron and the output neuron where $\beta_{j}={\left[\beta_{j1},\beta_{j2},\beta_{j3},...,\beta_{jm}\right]}^{T}$. Generally, the ELM network function is shown as [11]:

(10) $$t_{i}=\sum_{j=1}^{l}\beta_{j}g\left(\omega_{j}\cdot x_{i}+b_{j}\right),\;i=1,2,...,N;$$

where $j\in \left\{1,2,...,l\right\},\;\omega_{j}\cdot x_{i}$ denotes the inner product of $\omega_{j}$ and $x_{i}$; the activation function *g*(*x*) is the sigmoid function in the proposed method, which is formulated as:

(11) $$g\left(x\right)=\frac{1}{1+exp\left[-\left(\omega \cdot x+b\right)\right]}$$

Equation (10) can be simplified as the following simple form:

(12) Hβ=T

where:

(13) $$H={\left[\begin{array}{ccc} g(\omega_{1}\cdot x_{1}+b_{1}) & \cdots & g(\omega_{l}\cdot x_{1}+b_{l})\\ \vdots & \ddots & \vdots \\ g(\omega_{1}\cdot x_{N}+b_{1}) & \cdots & g(\omega_{l}\cdot x_{N}+b_{l}) \end{array}\right]}_{N\times l}$$

*H* is defined as the hidden layer output matrix of the network. *T* is the output matrix, and $T={\left[t_{1},t_{2},...,t_{N}\right]}^{T}$.

In the ELM algorithm, the input weights and hidden biases are chosen at random, and the output weights are calculated through the equation below:

(14) $$\beta =H^{+}T$$

where *H*^+^ is the Moore–Penrose (MP) generalized inverse of matrix *H*.

The learning procedure of ELM can be summarized as the four steps in Algorithm 1.
**Algorithm 1.** Extreme learning machine (ELM).1. Given a training set with *N* distinct examples $\Psi ={\left\{\left(x_{i},t_{i}\right)|x_{i}\in R^{n},t_{i}\in R^{m}\right\}}_{i=1}^{N}$, activation function *g*(*x*) and hidden neuron number l;2. Set input weights *ω* and hidden biases *b* from [–1,1] at random;3. Calculate the hidden layer output matrix *H* using matrix multiplication;4. Calculate the output weights $\beta =H^{+}T$ according to the Moore–Penrose generalized inverse.

### 4.2. The Framework of the ELM-Based Gravity Compensation Method

**Figure 5:**
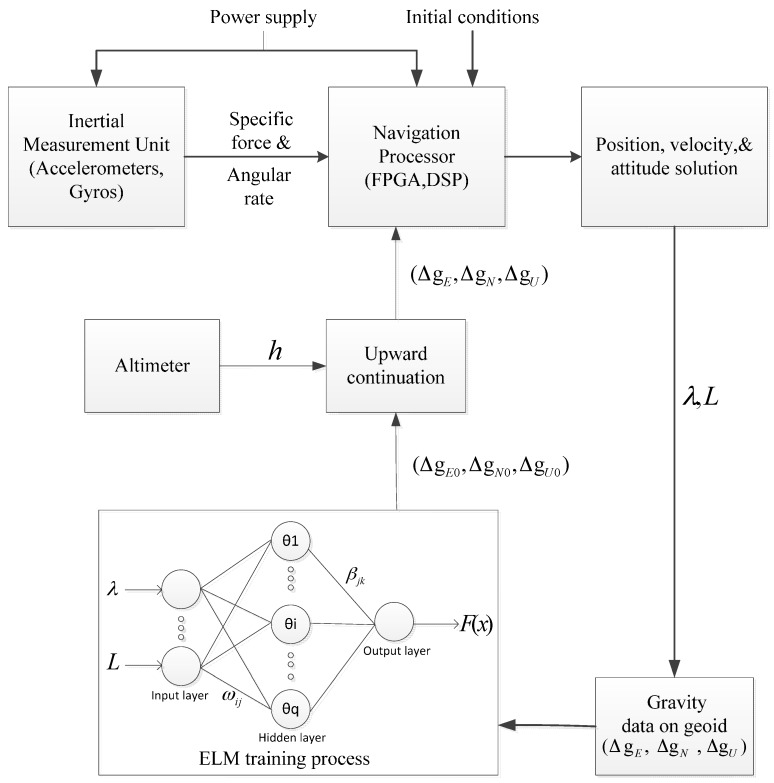
The framework of ELM-based gravity compensation method.

The framework of the ELM-based gravity disturbance compensation method is shown in Figure 5 [22,23].

The process of the ELM-based gravity disturbance compensation method is described as follows:

1. Get the INS position. Obtain the position value of the INS, longitude (λ) and latitude (L), through the calculation of INS.
2. Choose the gravity database. Search the adaptive gravity database (provided by Institute of Geodesy and Geophysics, Chinese Academy of Sciences) according to the position obtained by Step 1. The gravity disturbance is related to the correlation distances. This means too wide a training area will not improve the estimation result, and too small a training area will not include enough information for the estimation of the gravity disturbance. After many trainings, we found that the size of the training area set as 5′ × 5′ would have the best estimation result. Therefore, here, the gravity database is set as 5′ × 5′ and takes the position of the INS as the central point.
3. ELM training: Set λ, L as the inputs of the ELM algorithm, and obtain the gravity disturbance on the geoid ($\Delta g_{E0},\Delta g_{N0},\Delta g_{U0}$) through the training with the gravity database obtained by Step 2.
4. Upward continuation: Process the gravity disturbance with upward continuation to the height where the INS is. The height of the INS is obtained by the altimeter. In geographic engineering applications, the most practical upward continuation method is free air correction. The computational formula is described as follows [24]:
  (15) $$\Delta g=\Delta g_{0}-0.3086H$$
  where $\Delta g_{0}$ is the value of the gravity disturbance on the geoid, Δg is the value of the gravity disturbance where the INS is and *H* is the height value between the geoid and INS.
5. Compensate the gravity disturbance calculated by Step 4 in the INS error equations to restrain the error propagation caused by gravity disturbance.

## 5. Numerical Test

**Figure 6:**
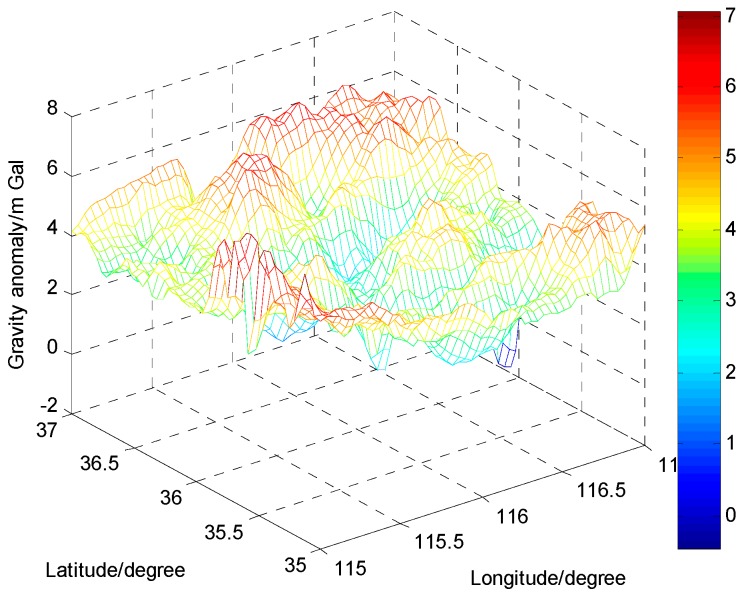
The gravity anomaly map in the Huabei plain area.

**Figure 7:**
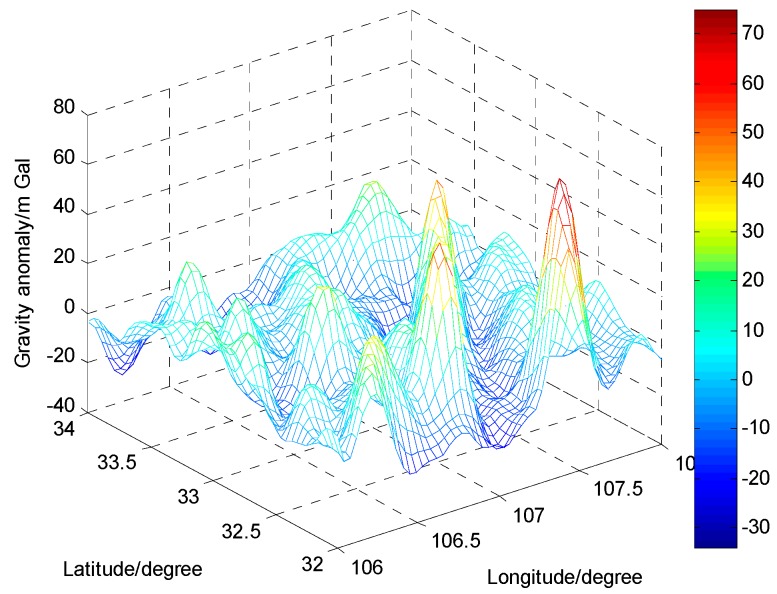
The gravity anomaly map in the Qinling mountain area.

To test the accuracy of the ELM-based gravity disturbance estimation method, two areas each with 50 gravity data points derived from the Institute of Geodesy and Geophysics, Chinese Academy of Sciences, are chosen as the application regions. Additionally, the resolution of the gravity database is 1′ × 1′. Region 1 is in the Huabei plain area in China where there is a mild gravity variation; Region 2 is in the Qinling mountain area in China where there is a rough gravity variation. The gravity anomaly maps in the two application regions are shown in Figure 6 and Figure 7. In each region, the gravity points are divided into two parts: 10 points are used as testing data; the other 40 points as training data.

The evaluation criterions chosen to evaluate the performance of the estimation methods are mean absolute error (MAE), mean radial error (MRE) and root mean square error (RMSE). MAE can reflect the possible estimation error range of estimation methods; MRE stands for the ensemble estimation error; and RMSE reflects the sensitivity and extreme value effect of the estimation results. The calculation formulae of these three evaluation criteria are described as follows [25,26]:

(16) $$MAE=\frac{1}{n}\sum_{i=1}^{n}ABS(X_{m,i}-X_{e,i})$$

(17) $$MRE=\frac{1}{n}\sum_{i=1}^{n}\left[ABS(X_{m,i}-X_{e,i})/X_{m,i}\right]$$

(18) $$RMSE=\sqrt{\frac{1}{n}\sum_{i=1}^{n}{\left(X_{m,i}-X_{e,i}\right)}^{2}}$$

where *n* is the number of the testing points and $X_{m,i}$, $X_{e,i}$ are the measured and estimated gravity disturbance values, respectively. ABS is the symbol of the absolute value function.

The performances of the different estimation methods applied in two test regions are listed in Table 2 and Table 3.

From Table 2 and Table 3, we can see that the ELM-based estimation method had a better performance than the other two methods, especially in the mountain area with rough topography. The RMSE of the ELM estimation method improved by 23% and 44% compared with the bilinear interpolation in the plain and mountain area, respectively. Additionally, for the other two variables of gravity disturbance, we had similar estimation results as the gravity anomaly. The estimation performances of the other two variables with the ELM method were better than the two traditional methods.

## 6. Experiment

**Figure 8:**
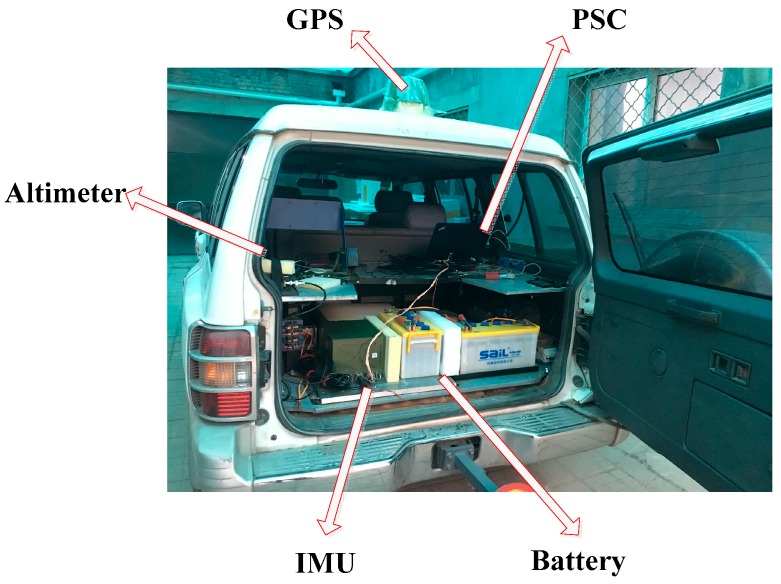
Field test device. PSC, processing computer system.

To further validate the proposed gravity compensation method, two field experiments were carried out in Shanxi province in China: one was in an urban road with a mild gravity variation, and the other one was in a mountain road with a fierce gravity variation. On the test car were carried a GPS receiver and the INS, which contained the inertial measurement unit (IMU) and the processing computer system (PSC). The PSC collects the GPS data and the IMU data for data storage, then calculates it for navigation. The altimeter and battery were also carried on the test car for measurement and electricity supply. The test car is shown in Figure 8. The performances of the inertial sensors and GPS are listed in Table 4. The resolution of the gravity database is 1′ × 1′ (provided by the Institute of Geodesy and Geophysics, Chinese Academy of Sciences).

**Figure 9:**
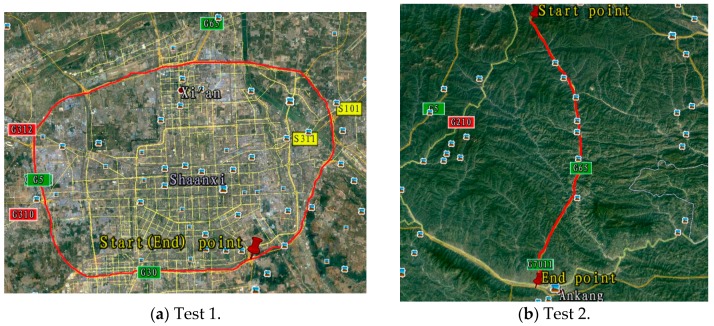
Field test trajectories on Google map.

**Figure 10:**
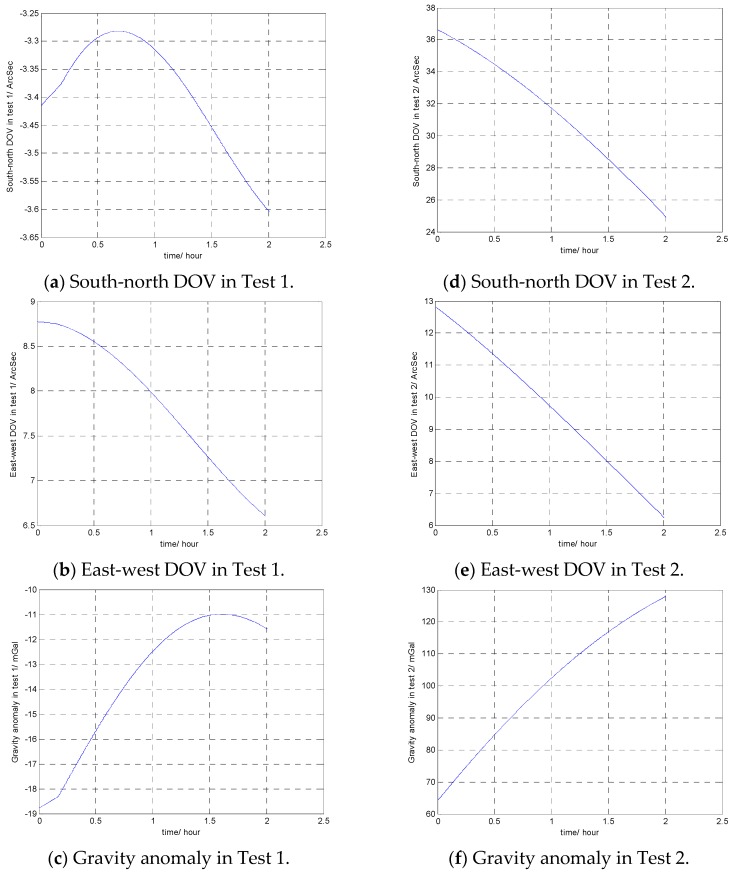
The gravity anomaly and deflections of the vertical (DOVs) of the two tests.

Field Test 1 was carried out in Xi’an city area in China, and field Test 2 was carried out in Qinling mountain area in China. The travel profiles, gravity anomalies and DOVs on the two trajectories are shown in Figure 9 and Figure 10.

**Figure 11:**
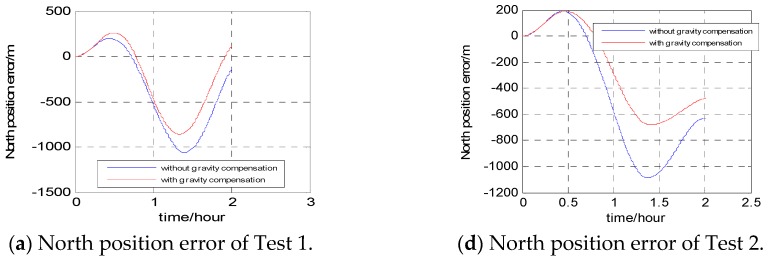

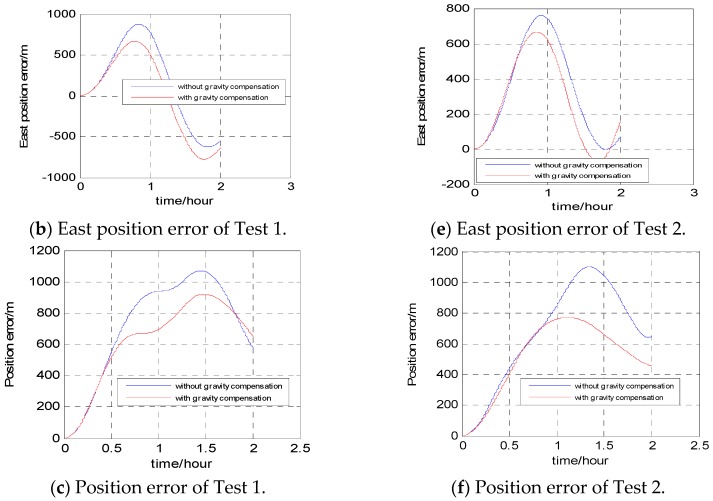
Position errors of the two tests.

The position results without gravity compensation and results with the proposed gravity compensation method are compared with the GPS result, as shown in Figure 11. In Figure 11a–c are the position results of Test 1, Figure 11d–f are the position results of Test 2.

Compared with the position results of GPS, the maximum position errors of two tests are listed in Table 5.

The results in Figure 11 and Table 5 show that the proposed gravity compensation method can both improve the positioning accuracy in the two tests, especially in Test 2 with the rough topography, because in Test 2 along the trajectory, the value of the gravity disturbance is much bigger and has a wider variation range. According to the characteristics of the error propagation in free-INS, the error caused by the gravity disturbance increases with time. This can be verified in Figure 11. The errors shown in Figure 11 are similar for both methods (without and with the gravity compensation) at the beginning of the experiment, but they diverge in the later stages. During the 2-h field experiment, the maximum value of position error improved by 13% and 29%, respectively, in Tests 1 and 2, when the navigation scheme was compensated by the proposed gravity compensation method. Therefore, the effectiveness of the proposed gravity compensation method is verified in the field experiments.

## 7. Conclusions

In this paper, a novel gravity compensation method for high-precision INS is proposed, which uses the ELM-based estimation method to obtain the optimal gravity disturbance on the track based on measured gravity data on the geoid, then processes gravity disturbance on the geoid to the height where INS has an upward continuation. Finally, the estimated gravity disturbance on the trajectory is compensated into the INS error equations to restrain the error propagation in INS. The accuracy of the proposed estimation method was evaluated using the numerical tests, which divided the test area into two parts, one was in the plain area and the other in the mountain area. The estimation results showed that the ELM-based estimation method had a better performance than the other two methods, especially in the mountain area with rough topography. The RMSE of the ELM-based estimation method improved by 23% and 44% compared with the bilinear interpolation in the plain and mountain area, respectively. To further verify the performance of the proposed gravity compensation method, two field experiments were carried out with a high-precision INS, and the experiment results showed that the positioning accuracy improved by 13% and 29%, respectively, in the plain area and mountain area with the proposed gravity compensation method. It should be noted that in this work, the experiment was conducted only in cars at a relatively low speed. In the future, a flight experiment with relative high travel speed should be carried out to further verify the efficiency of the proposed gravity compensation method.

## Figures and Tables

**Table 1:** The north component of gravity and the amplitude of the north position error corresponding to the gravity vertical deflection.

	ζ = 2 s	ζ = 8 s	ζ = 15 s	ζ = 25 s	ζ = 35 s
$\Delta g_{N}$ (m Gal *)	9	38	71	118	166
North position error (m)	117	494	923	1534	2160

* 1 m Gal = 1 × 10^−5^ m/s^2^.

**Table 2:** Performance of the estimation methods in test Region 1. MRE, mean radial error; IDW, inverse distance weighted method.

Evaluation Criterion	Estimation Methods		
	IDW	Bilinear Interpolation	ELM
MAE	0.157	0.138	0.098
MRE	0.059	0.058	0.032
RMSE	0.285	0.279	0.213

**Table 3:** Performance of the estimation methods in test Region 2.

Evaluation Criterion	Estimation Methods		
	IDW	Bilinear Interpolation	ELM
MAE	0.228	0.203	0.128
MRE	0.076	0.056	0.041
RMSE	0.367	0.314	0.193

**Table 4:** Performance of the sensors for the experiment.

Sensors Types	Characteristics	Magnitude (1 σ)
Gyroscope	Constant bias	0.003°/h
Accelerometer	Constant bias	10 μg
GPS velocity	Horizontal error	0.03 m/s
	Height error	0.05 m/s
GPS position	Horizontal error	2 m
	Height error	5 m
Altimeter	Measurement error	±5 m
	Measurement resolution	0.1 m

**Table 5:** The maximum value of position error (m) compared with the GPS result.

	Without Gravity Compensation	With Gravity Compensation	Position Improvement
Test 1	1050	913	137 (13%)
Test 2	1120	790	330 (29%)
